# Associations between long-term exposures to airborne PM_2.5_ components and mortality in Massachusetts: mixture analysis exploration

**DOI:** 10.1186/s12940-022-00907-2

**Published:** 2022-10-11

**Authors:** Tingfan Jin, Heresh Amini, Anna Kosheleva, Mahdieh Danesh Yazdi, Yaguang Wei, Edgar Castro, Qian Di, Liuhua Shi, Joel Schwartz

**Affiliations:** 1 Department of Environmental Health, Harvard T.H. Chan School of Public Health, Boston, MA USA; 2 Department of Public Health, University of Copenhagen, Copenhagen, Denmark; 3 Department of Family, Population, & Preventive Medicine, Program in Public Health, Stony Brook University, New York, NY USA; 4 Vanke School of Public Health, Tsinghua University, Beijing, China; 5 Gangarosa Department of Environmental Health, Rollins School of Public Health, Emory University, Atlanta, GA USA

**Keywords:** Air pollution, Particle components, Weighted quantile sum regression

## Abstract

**Background:**

Numerous studies have documented PM_2.5_’s links with adverse health outcomes. Comparatively fewer studies have evaluated specific PM_2.5_ components. The lack of exposure measurements and high correlation among different PM_2.5_ components are two limitations.

**Methods:**

We applied a novel exposure prediction model to obtain annual Census tract-level concentrations of 15 PM_2.5_ components (Zn, V, Si, Pb, Ni, K, Fe, Cu, Ca, Br, SO_4_^2−^, NO_3_^−^, NH_4_^+^, OC, EC) in Massachusetts from 2000 to 2015, to which we matched geocoded deaths. All non-accidental mortality, cardiovascular mortality, and respiratory mortality were examined for the population aged 18 or over. Weighted quantile sum (WQS) regression models were used to examine the cumulative associations between PM_2.5_ components mixture and outcomes and each component’s contributions to the cumulative associations. We have fit WQS models on 15 PM_2.5_ components and a priori identified source groups (heavy fuel oil combustion, biomass burning, crustal matter, non-tailpipe traffic source, tailpipe traffic source, secondary particles from power plants, secondary particles from agriculture, unclear source) for the 15 PM_2.5_ components. Total PM_2.5_ mass analysis and single component associations were also conducted through quasi-Poisson regression models.

**Results:**

Positive cumulative associations between the components mixture and all three outcomes were observed from the WQS models. Components with large contribution to the cumulative associations included K, OC, and Fe. Biomass burning, traffic emissions, and secondary particles from power plants were identified as important source contributing to the cumulative associations. Mortality rate ratios for cardiovascular mortality were of greater magnitude than all non-accidental mortality and respiratory mortality, which is also observed in cumulative associations estimated from WQS, total PM_2.5_ mass analysis, and single component associations.

**Conclusion:**

We have found positive associations between the mixture of 15 PM_2.5_ components and all non-accidental mortality, cardiovascular mortality, and respiratory mortality. Among these components, Fe, K, and OC have been identified as having important contribution to the cumulative associations. The WQS results also suggests potential source effects from biomass burning, traffic emissions, and secondary particles from power plants.

**Supplementary Information:**

The online version contains supplementary material available at 10.1186/s12940-022-00907-2.

## Background

Numerous studies have documented the associations between both short-term [1–6] and long-term [4, 7–15] exposures to fine particulate matter (PM_2.5_) and adverse health outcomes across different populations, especially on non-accidental mortality [4, 6–9, 11–15]. In the most updated Integrated Science Assessment (ISA) for Particulate Matter, the EPA has concluded a causal relationship between long-term PM_2.5_ exposure and mortality exists [16]. Determining the relative toxicity of PM_2.5_ components could be very helpful in facilitating PM_2.5_ pollution control efforts.

However, fewer studies have evaluated the associations between long-term exposure to PM_2.5_ components and mortality as well as the relative contributions of different components to the cumulative associations between PM_2.5_ and mortality. Inconsistent findings on the associations between PM_2.5_ components and mortality risks have been reported in previous studies [17–19]. A key limitation in examining PM_2.5_ components is the general lack of high-resolution exposure measurements. Some studies have obtained data from fixed monitoring sites [20–22]. However, compared to the total PM_2.5_ mass, there are fewer monitors for PM_2.5_ components, and sometimes the spatial heterogeneity of some components, such as elemental carbon, is much greater than for overall PM_2.5_. Therefore, those studies were limited in capturing the spatial variation of PM_2.5_ components concentrations, which is critical to a study of long-term exposure on large population and at large geographic scale. More recently, exposure prediction models with higher spatial resolution have been used as an alternative, including chemical transport models [23–25] and land use regression models [17–19, 26, 27]. However, many of these models have relatively moderate prediction accuracies and spatial resolutions. Therefore, it is worthwhile to use a model with a higher spatial resolution and out-of-sample cross-validated prediction accuracy to better capture PM_2.5_ component exposure and further evaluate their links with adverse health outcomes.

Another key problem to study the potential associations between PM_2.5_ components and adverse health outcomes is the high correlation among different particle components which have been reported by some previous studies [21, 27, 28]. There are serious collinearity issues in comparing each component’s contribution to the outcome using traditional regression methods. Weighted quantile sum (WQS) regression is one approach to estimate the cumulative associations between multiple components and adverse health outcomes and identify the “bad actors” (considered as most toxic components among all) when there are collinearity issues [29, 30]. The WQS approach has already been applied in previous studies investigating the links between complex environmental mixtures and health outcomes[31, 32].

In this study, we have applied novel exposure prediction models which have generated high-resolution PM_2.5_ component concentration data across the contiguous U.S. to capture the exposure in Massachusetts. The exposure models combined land use, satellite observations, traffic, meteorological variables, chemical transport model output, and monitoring data for 15 PM_2.5_ components. We evaluated the associations between long-term exposures to PM_2.5_ components mixture and all non-accidental mortality, cardiovascular mortality, and respiratory mortality. The WQS regression models were fit to examine the cumulative associations and each component’s contribution to the cumulative associations. We also conducted additional analysis based on the a priori identified source categories for the PM_2.5_ components, and the total PM_2.5_ mass analysis with single component associations examinations as supplemental secondary analysis.

## Methods

This study was conducted at the Census tract level in the state of Massachusetts. The study period was from 2000 to 2015. The following exposure data, mortality data, and covariates data were collected and processed within this area and during this period.

### Exposure data

15 PM_2.5_ components were selected in our study: zinc (Zn), vanadium (V), silicon (Si), lead (Pb), nickel (Ni), potassium (K), iron (Fe), copper (Cu), calcium (Ca), bromine (Br), sulfate (SO_4_^2−^), nitrate (NO_3_^−^), ammonium (NH_4_^+^), organic carbon (OC), and elemental carbon (EC). Five of them were the major mass contributors to total PM_2.5_: SO_4_^2−^, NO_3_^−^, NH_4_^+^, OC, and EC, which will be referred to as “major components” in the following content. The other ten are trace elements. One reason to select these components was the number of monitors available to train the models. Another important reason to select these components is the relative toxicity of them. We chose to estimate the concentrations of 10 trace elements as well as the major components because there are evidence showing the trace elements are important for PM_2.5_ toxicity despite their low mass concentrations [33–40]. Moreover, the 10 trace elements represent various periodic table groups (groups 1 (K), 2 (Ca), 5 (V), 8 (Fe), 10 (Ni), 11 (Cu), 12 (Zn), 14 (Si and Pb), and 17 (Br)) as elements in the same group have similar physical or chemical characteristics.

We have generated annual mean predictions for the 15 PM_2.5_ components’ concentrations across the contiguous U.S. at a 50 m × 50 m spatial resolution in urban areas and a 1 km × 1 km spatial resolution in non-urban areas by year over the study period [41]. Specifically, we collected daily mean PM_2.5_ component data from 987 monitoring sites across the United States (where 69 are located in Massachusetts). 166 predictors were used for modeling, including time and geography information (year, latitude, longitude, elevation), satellite observation data (vegetation, water index, nighttime lights, CO and CH4, aerosol optical depth, etc.), meteorological data (temperature, humidity, wind velocity and direction, planetary boundary layer height, etc.), emitting/surrogate of emission sources (distance to power plants, distance to highways, traffic counts, burning index, etc.), and many others.

In non-urban areas, five machine-learning algorithms were fit to predict each component and then six super-learners and an ensemble weighted averaging model (ENWA) were applied to integrate predictions from the individual machine-learning algorithms. In urban areas, due to the computational limitations, we fit only three machine-learning algorithms and integrated predictions using four super-learners and an ENWA. The super-learner or ENWA with best performance was chosen as the final model, which varied by PM_2.5_ component. With inclusion of a large number of monitoring sites across the US and many new predictors, this approach yielded excellent model performance at high spatial resolution, with out-of-sample validation R^2^ for individual components ranging from 0.821 (Br in urban areas) to 0.957 (SO_4_^2−^ in non-urban areas) [42, 43]. The root-mean-square error (RMSEs) calculated from regressing the monitored values against the predicted values specific in Massachusetts are presented in the supplemental materials (Table S1 in Additional File 1).

Each Census tract was matched to all the grid cells whose centroids were in it. We then averaged these grid cell-level predictions for each component at the Census tract level for each calendar year and assigned the Census tract-level annual means to each subject. Figure S1a and S1b in Additional File 1 shows the Census tract-level mean concentrations for each component in Massachusetts. Table S2 in Additional File 1 shows how the mean annual average concentrations across all census tracts in Massachusetts of the 15 PM_2.5_ components vary from 2000 to 2015.

In addition to the 15 PM_2.5_ components data, we also obtained the daily PM_2.5_ (24-hour mean) predictions at 1 km×1 km spatial resolution from 2000 to 2015 in the state of Massachusetts from a spatial-temporal ensemble model integrating three machine learning algorithms (neural networks, random forest, gradient boosting) [44]. We then calculated the annual average PM_2.5_ concentrations for each 1 km×1 km grid from 2000 to 2015 and aggregated them to the census tract level like the PM_2.5_ components concentrations. These PM_2.5_ concentrations were used for total PM_2.5_ mass analyses supporting the interpretation of PM_2.5_ components analyses results.

### Mortality Data

We obtained the records of all deaths for years 2000–2015 from the Massachusetts Department of Public Health. These mortality records included the residential address (geocoded to longitude and latitude), age, sex, race, date of death, and cause of death (Underlying Cause of Death, ICD-10 codes). We restricted our analysis to non-accidental mortality (ICD-10 codes A-R) and dropped mortality records with missing age or residential address and restricted our study to decedents with age ≥ 18 years. This procedure left 787,352 deaths remaining in the final dataset. We aggregated the deaths by Census tract and year. We also calculated yearly counts of deaths from cardiovascular diseases (ICD-10 codes I00 to I99) and deaths from respiratory diseases (ICD-10 codes J00 to J99) based on the cause of death information.

### Covariates

***Meteorological Data*** – We obtained the daily maximum and minimum relative humidity and temperature data from gridMET at a 4 km × 4 km spatial resolution (01/01/2000–12/31/2015) and calculated the daily mean values by averaging the maximum and minimum values [45]. We then calculated the annual average relative humidity and temperature. Each Census tract was linked to its nearest grid cell based on the distances between the centroids of the census tract and the grid cell, then the meteorological measures were assigned accordingly.

***Census Data*** – Socioeconomic and demographic data were obtained from the 2000 and 2010 Decennial Censuses, and the 2009–2015 American Community Survey (ACS) 5-year estimate data retrieved from the IPUMS data service [46]. We pre-specified and collected data of potential confounders at the Census tract level including median household income, population density, proportion female, proportion African American, proportion Hispanic or Latino, proportion aged 65 or older, proportion whose income was below poverty level, and proportion with at least a high school degree or equivalent. We also obtained the total population with aged 18 or older for each Census tract. For years 2001–2008, the data were interpolated linearly from the observed data for each variable separately, using year as the sole predictor. This interpolation was applied to all census variables included in our analysis.

***Lung Cancer Admission Rate Data*** – We computed the ZIP code-level lung cancer hospitalization rate data from the MEDPAR files of the Center for Medicare and Medicaid Services. This lung cancer hospitalization rate variable was used as a surrogate for cumulative smoking. Each Census tract was first matched to the ZIP Code Tabulation Area (ZCTA) with maximum overlap, and then matched to the corresponding ZIP code.

### Statistical methods

We had three outcomes of interest: all non-accidental mortality, cardiovascular mortality, and respiratory mortality. The main exposures of interest were the aforementioned 15 PM_2.5_ components. The covariates selected for confounder control included the meteorological variables, socioeconomic and demographic variables, and lung cancer admission rates.

Our main analysis is assessing the cumulative associations between 15 PM_2.5_ components and three different mortality outcomes by fitting weighted quantile sum (WQS) regression models. WQS is a supervised mixture analysis methods treating multiple pollutants as a mixture to which people were simultaneously exposed. In WQS, two parts of calculation are conducted. First, WQS estimates a composite index summarizing the pollutant mixture. This index is a weighted sum of each pollutant’s quantiles (WQS term) based on each pollutant’s relevance to the outcome being examined. Second, the WQS model estimates the cumulative association between the mixture and outcome by fitting a multivariate regression model with the WQS term as the exposure metric.

One advantage of WQS is it could reduce the impact of collinearity and extreme values from highly correlated pollutants in estimating the cumulative associations. In this study, we have 15 correlated PM_2.5_ components (Figure S2a and S2b in Additional File 1). Moreover, we could also interpret each pollutant’s contribution to the cumulative associations through the weights assigned to them in the WQS term [29]. The estimated weights could vary across different outcomes examined in WQS models.

For each outcome of interest (i.e., all non-accidental mortality, cardiovascular mortality, respiratory mortality), a separate WQS regression model was fit. More detailed explanation on how the WQS models were fit could be found in Paragraphs S1 in Additional File 1.Generally, the dataset would first be split into a training set (to estimate the WQS index and weights) and a validation set (to estimate the cumulative association), which we set as 50:50. We then scored the components concentrations into deciles and generated 250 bootstrap samples of the training set. In each bootstrap sample, a separate set of weights was estimated. Only the positive or null associations between PM_2.5_ components and mortality were assessed in our study since we know of no evidence suggesting any of these components is beneficial for health through inhalation. Thus, the final weights of each component were estimated by averaging the weight estimated from the bootstrap samples whose results showed a positive or null association between PM_2.5_ components and mortality. The final WQS index could be presented as $\sum _{1}^{15}{w}_{i}{q}_{i}$ where ${w}_{i}$ is the weight for the ith component concentration and ${q}_{i}$ is the quantile score for that component concentration for that observation.

Then, the cumulative association between the mixture and outcome was estimated in the validation set through multivariate quasi-Poisson regression models. Several previous studies have used the Poisson regression models to examine the health effects of long-term air pollution exposures[47–51]. In our study, to address the overdispersion issue, we fit quasi-Poisson regression models all over the analyses. The formula for the regression models could be presented as:

$$log\left(count\right)={\theta }_{0}+{\theta }_{1}\bullet WQS+{{\varvec{\theta }}_{2}}^{\varvec{{\prime }}}\bullet \varvec{X}+offset\left(log\right(population\left)\right)$$

In this formula, *count* was the annual number of deaths; *WQS* was the WQS term, the exposure of interest; ***X*** were the covariates including the meteorological data, lung cancer admission data, socioeconomic data, and demographic data; and *offset(log(population))* was the offset term for mortality rate estimation. In WQS, each ${w}_{i}$ was constrained between 0 and 1, and the sum of weights $\sum _{1}^{15}{w}_{i}$ was constrained to be 1. Therefore, one unit increase in final WQS index could be treated as approximately one decile increase in all components, and we interpreted the cumulative association estimates as the mortality rate ratio associated with this increase.

We also conducted several additional analyses of WQS models, and two additional sets of WQS models were fit. In each additional set, one separate WQS models was fit for each outcome of interest.

First, we noticed that there is evidence suggesting that interpreting the sum of the weights for a group of pollutants could be more reasonable than interpreting the weights for the single pollutants with highly correlated pollutants [29]. We have identified 8 source groups a priori: heavy fuel oil combustion, biomass burning, crustal matter, non-tailpipe traffic source, tailpipe traffic source, secondary particles from power plants, secondary particles from agriculture, and unknow source. We grouped the 15 PM_2.5_ components into each category and calculated the sums of the weights of components for each source group to have a comparison among different sources.

Second, we noticed that there was a clear highly correlated cluster of 14 components except K in our study(Figure S2a and S2b in Additional File 1) and a cluster of highly correlated pollutants may result in underestimated weights in WQS models [29]. Therefore, it was possible that K may “steal” the weights from other components. Hence, we also fit an additional set of WQS models without K to see how the results would change against this exclusion.

Finally, to further support the interpretation of we also fit a WQS models on the source groups aforementioned rather than directly on the 15 PM_2.5_ components. To do so, we first calculated the total mass of each source groups by summing the components mass of each group, and then fit WQS models taking the source groups as the individual pollutant in WQS models, with all other model settings and parameters the same as the components WQS models.

In addition to the WQS models, we conducted a total PM_2.5_ mass analysis through a quasi-Poisson regression model with PM_2.5_ as the exposure term. Moreover, we also fit single PM_2.5_ component models to see the individual-by-individual associations with the outcomes. In these quasi-Poisson models, penalized splines with penalty parameter chosen using restricted maximum likelihood estimation were used to adjust for potentially nonlinear confounding. In the single PM_2.5_ component models, confidence intervals were further adjusted for multiple comparisons using the Bonferroni correction.

All the data work and statistical analysis were conducted using R 4.1.1, and all WQS models were fit using gWQS 3.0.4 package [52].

## Results

Of the 787,352 non-accidental deaths in the final dataset, 54.2% were female and 93.1% were white. The average age of death was 77.7 years. There were 263,157 deaths (33.4%) from cardiovascular diseases and 87,197 deaths (11.1%) from respiratory diseases. More detailed summary statistics are presented in Table 1.

**Table 1:** Summary characteristics of mortality records in the final dataset

Characteristics	n (%)
**Total**	787,352 (100)
**Gender**	
Male	360,464 (45.8)
Female	426,873 (54.2)
Missing	15 (0.00019)
**Race**	
White	733,320 (93.1)
Black	31,387 (4.0)
Other	22,558 (2.9)
Missing	87 (0.01)
**Cause of Death**	
Cardiovascular Diseases	263,157 (33.4)
Respiratory Diseases	87,197 (11.1)
Other Non-accidental	436,998 (55.5)
**Mean Age (Standard Deviation)**	77.68 (14.15)

Table 2 summarizes the characteristics of the Census tract-level annual mean PM_2.5_ components’ concentrations, relative humidity, and temperature. SO_4_^2−^ contributed the most to the total mixture mass, with a mean annual concentration of 2.21 µg/m^3^. Organic carbon also contributed a large portion with a mean annual concentration of 1.72 µg/m^3^. For the trace element components, Si, Fe, and K had the highest contribution to the total mixture mass with mean annual concentrations of 54.34 ng/m^3^, 50.80 ng/m^3^, and 47.81 ng/m^3^, respectively. The mean annual relative humidity was 64.5% and the mean annual temperature was 10.1 °C.

**Table 2:** Summary characteristics of particle component concentration, relative humidity, and temperature

Variable	Median	Mean	IQR	S.D.	75th	95th	Max
*PM*_*2.5*_ *Major Components*							
SO_4_^2−^ (µg/m^3^)	2.36	2.21	1.41	0.75	2.87	3.25	3.76
NO_3_^−^ (µg/m^3^)	0.80	0.82	0.26	0.18	0.94	1.15	1.57
NH_4_^+^ (µg/m^3^)	0.75	0.76	0.45	0.25	0.96	1.17	1.49
OC (µg/m^3^)	1.64	1.72	0.47	0.39	1.92	2.51	3.56
EC (µg/m^3^)	0.45	0.48	0.16	0.14	0.55	0.76	1.23
*PM*_*2.5*_ *Trace Elements*							
Zn (ng/m^3^)	9.29	9.49	3.82	2.72	11.36	14.12	19.18
V (ng/m^3^)	1.43	1.70	2.00	1.29	2.54	4.15	6.84
K (ng/m^3^)	47.63	47.81	6.20	4.77	50.87	55.83	67.21
Si (ng/m^3^)	51.15	54.34	17.63	13.79	61.47	83.93	107.14
Pb (ng/m^3^)	1.96	2.39	1.77	1.07	3.30	4.33	5.52
Ni (ng/m^3^)	0.98	1.14	0.86	0.63	1.52	2.41	3.03
Fe (ng/m^3^)	49.72	50.80	22.12	17.20	61.01	82.70	124.43
Cu (ng/m^3^)	2.47	2.66	1.30	1.15	3.18	5.03	9.92
Ca (ng/m^3^)	22.68	23.30	6.94	5.59	26.36	34.00	50.17
Br (ng/m^3^)	2.63	2.63	0.41	0.32	2.83	3.15	3.68
*Total PM*_*2.5*_ *Mass*							
PM_2.5_ (µg/m^3^)	8.75	8.73	2.80	1.94	10.08	11.96	16.39
*Meteorological variables*							
Relative humidity (%)	64.44	64.53	3.28	2.91	66.03	69.74	77.32
Temperature (℃)	10.09	10.09	1.36	1.05	10.83	11.72	12.86

Abbreviations: IQR for interquartile range; S.D. for standard deviation; 75th for 75th percentile; 95th for 95th percentile; Max for maximum value; SO_4_^2−^ for sulfate; NO_3_^−^ for nitrate; NH_4_^+^ for ammonium; OC for organic carbon; EC for elemental carbon; Zn for zinc; V or vanadium; K for potassium; Si for silicon; Pb for lead; Ni for nickel; Fe for iron; Cu for copper; Ca for calcium; Br for bromine

The correlations between PM_2.5_ components ranged from 0.04 for K-Si and K-Fe to 0.87 for NH_4_^+^-SO_4_^2−^. The correlations between K and other components were generally lower than the correlations among other 14 components. The detailed correlation matrix was presented in Figure S2a and S2b in Additional File 1.

### WQS Models on 15 PM_2.5_ components

Table 3 summarizes the cumulative association estimates from WQS models on 15 PM_2.5_ components. We found positive associations between the PM_2.5_ components mixture and all three outcomes. The mortality rate ratio for cardiovascular mortality (RR = 1.0533, 95% CI 1.0486–1.0581 per approximately 1 decile increase in all PM_2.5_ components) was of greater magnitude than respiratory (RR = 1.0460, 95% CI 1.0392–1.0529) and all non-accidental mortality (RR = 1.0303, 95% CI 1.0265–1.0341).

**Table 3:** Cumulative associations between PM_2.5_ components and mortality estimated from WQS models^a^

Cause-specific Mortality	Mortality Rate Ratio
*WQS Models on all 15 PM*_*2.5*_ *components*	
All Non-accidental Mortality	1.0303 (1.0265, 1.0341)
Cardiovascular mortality	1.0533 (1.0486, 1.0581)
Respiratory mortality	1.0460 (1.0392, 1.0529)
*WQS Models on the source groups identified for the 15 PM*_*2.5*_ *components*	
All Non-accidental Mortality	1.0277 (1.0241, 1.0313)
Cardiovascular mortality	1.0488 (1.0444, 1.0532)
Respiratory mortality	1.0435 (1.0368, 1.0504)

^a^The mortality rate ratios are per 1 unit increase in the WQS index, which could be interpreted as approximately 1 decile increase in all PM_2.5_ components or the source groups identified for the PM_2.5_ components

**Fig. 1:**
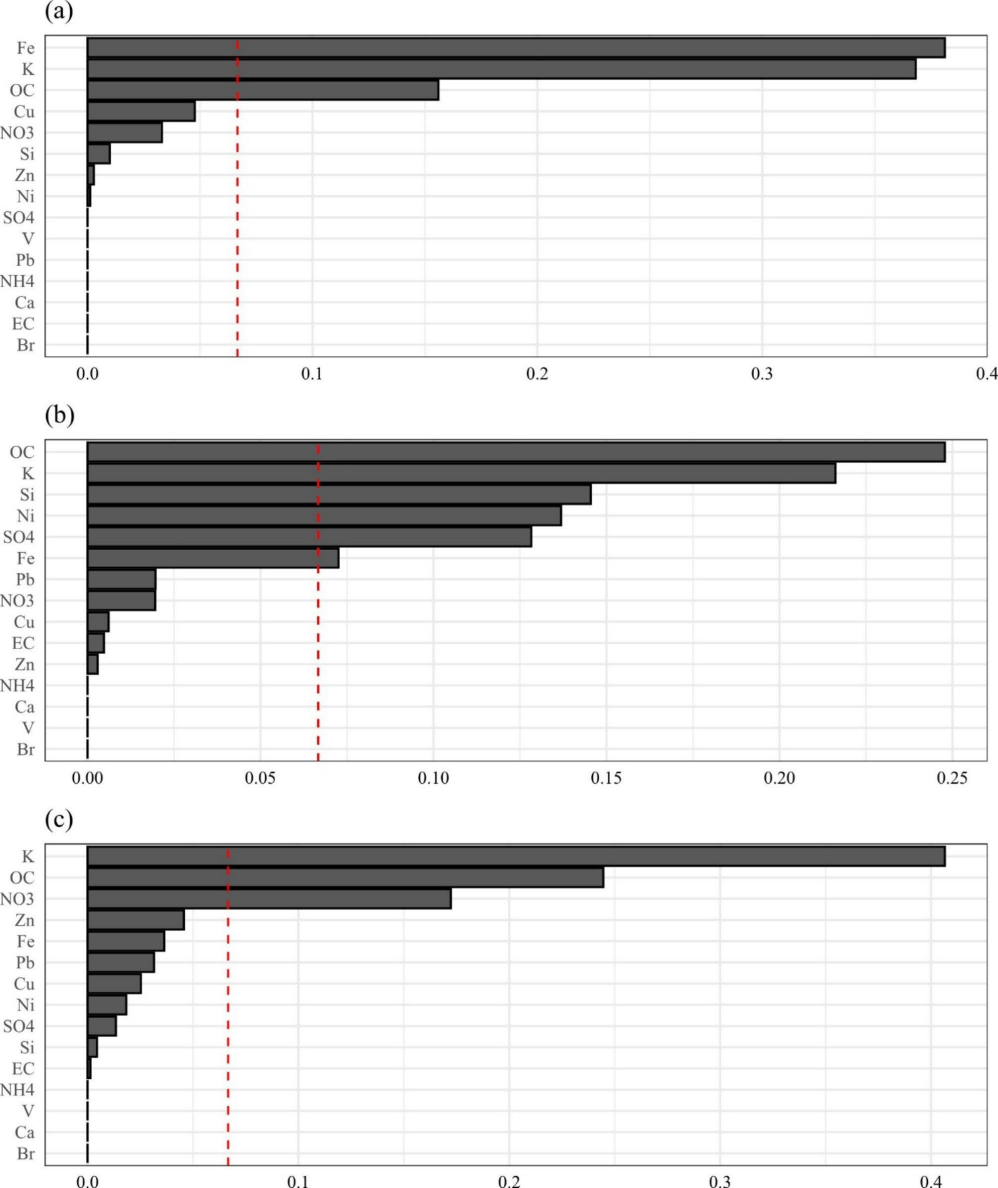
The weights assigned to each PM_2.5_ component from all-component WQS models. (a): all non-accidental mortality; (b): cardiovascular diseases related mortality; (c): respiratory diseases related mortality; red dash line: reciprocal of the number of PM_2.5_ components in the mixture

Figure 1 summarizes the weights of each component contributing to the associations with three outcomes. K and OC were found to have large weights to all three outcomes. Fe was found to have large weights to all non-accidental mortality and cardiovascular mortality. Besides, we also found large weights for Si, Ni, and SO_4_^2−^ to cardiovascular mortality and for NO_3_^−^ to respiratory mortality.

For the component mixture without K, we still found positive associations with all three outcomes and similar patterns across different causes. However, the mortality rate ratios were of less magnitude after dropping K from the mixture (Table S3 in Additional File 1). The orders of other components were relatively robust against to the dropping of K from the mixture. The components having large weights when including K in the components generally still had large weights when we dropped K (Figure S3 in Additional File 1).

### Sums of weights for source groups

The PM_2.5_ components grouped by a priori identified source categories and the sum weights for each source group are presented in Table 4. We have identified 8 source groups. The source groups with high sum weights could be identified as important source groups contributing to the cumulative association. Since OC has been assigned to both tailpipe traffic source and secondary particles (power plants), we have made an arbitrary split of OC’s weights giving 50% to each group.

**Table 4:** Source groups identified for PM_2.5_ components and WQS estimated weights assigned for the source groups

Sources	PM2.5 Components	All-cause	Cardiovascular Mortality	Respiratory Mortality
*Weights estimated from WQS models on all 15 PM*_*2.5*_ *components, sum the weights for identified source groups*				
G1: Heavy fuel oil combustion	V, Ni	0.0012	0.1369	0.0184
G2: Biomass burning	K	0.3682	0.2162	0.4065
G3: Crustal matter	Si, Ca	0.0099	0.1454	0.0045
G4: Non-tailpipe traffic source	Zn, Pb, Fe, Cu	0.4316	0.1012	0.1389
G5: Tailpipe traffic source^a^	EC, OC	0.0780	0.1286	0.1237
G6: Secondary particles, power plants^a^	SO_4_^2−^, NO_3_^−^, OC	0.1111	0.2717	0.3080
G7: Secondary particles, agriculture	NH4^+^	1.3×10^− 8^	3.2×10^− 8^	6.1×10^− 9^
G8: Unclear source	Br	8.8×10^− 9^	1.8×10^− 9^	1.8×10^− 9^
*Weights estimated from WQS models on the identified source groups*				
G1: Heavy fuel oil combustion	V, Ni	1.4×10^− 6^	0.0021	0.0002
G2: Biomass burning	K	0.4073	0.2342	0.4511
G3: Crustal matter	Si, Ca	0.0006	0.1069	0.0010
G4: Non-tailpipe traffic source	Zn, Pb, Fe, Cu	0.4472	0.0792	0.1586
G5: Tailpipe traffic source^a^	EC, OC	0.1445	0.2678	0.2674
G6: Secondary particles, power plants^a^	SO_4_^2−^, NO_3_^−^, OC	0.0004	0.3096	0.1209
G7: Secondary particles, agriculture	NH4^+^	6.7×10^− 6^	0.0002	0.0006
G8: Unclear source	Br	6.9×10^− 7^	6.9×10^− 10^	0.0002

Abbreviations: All-cause for all non-accidental mortality, Cardiovascular Mortality for cardiovascular diseases related mortality; Respiratory Mortality for respiratory diseases related mortality

^a^We have made an arbitrary split of OC between tailpipe traffic source and secondary particles (power plants): for the sums of the weights, 50% of the OC weights were summed into tailpipe traffic source, and the other 50% were summed into secondary particles (power plants) source; for the WQS models on source groups, 50% of the OC mass were summed into tailpipe traffic source, and the other 50% were summed into secondary particles (power plants) source

We can see that for all non-accidental mortality, non-tailpipe traffic source and biomass burning had the highest weights. Non-tailpipe and tailpipe traffic sources together consisted of over 50% of the total weights, indicating a strong contribution from traffic pollution to all non-accidental mortality. For respiratory mortality, we found highest weight for biomass burning, with secondary particles from power plants and the two traffic source groups also having large weights. The weights for cardiovascular mortality were more evenly distributed, with the weight for secondary particles from power plants as the highest. Biomass burning had the second highest weights, with traffic sources, heavy fuel oil combustion, and crustal matter also having weights over 0.1.

### WQS Models on source groups

The cumulative association estimates from WQS models on source groups are also presented in Table 3. We have also made an arbitrary split of OC’s mass giving 50% to each group of tailpipe traffic source and secondary particles (power plants). Similar to WQS models on PM_2.5_ components, we also found positive associations between the mixture and all three outcomes, with mortality rate ratio for cardiovascular mortality (RR = 1.0488, 95% CI 1.0444–1.0532 per approximately 1 decile increase in all PM_2.5_ components) of greater magnitude than respiratory (RR = 1.0435, 95% CI 1.0368–1.0504) and all non-accidental mortality (RR = 1.0277, 95% CI 1.0241–1.0313). The magnitudes of the mortality rate ratios estimated from WQS models on source groups were comparable to those estimated from WQS models on PM_2.5_ components.

**Fig. 2:**
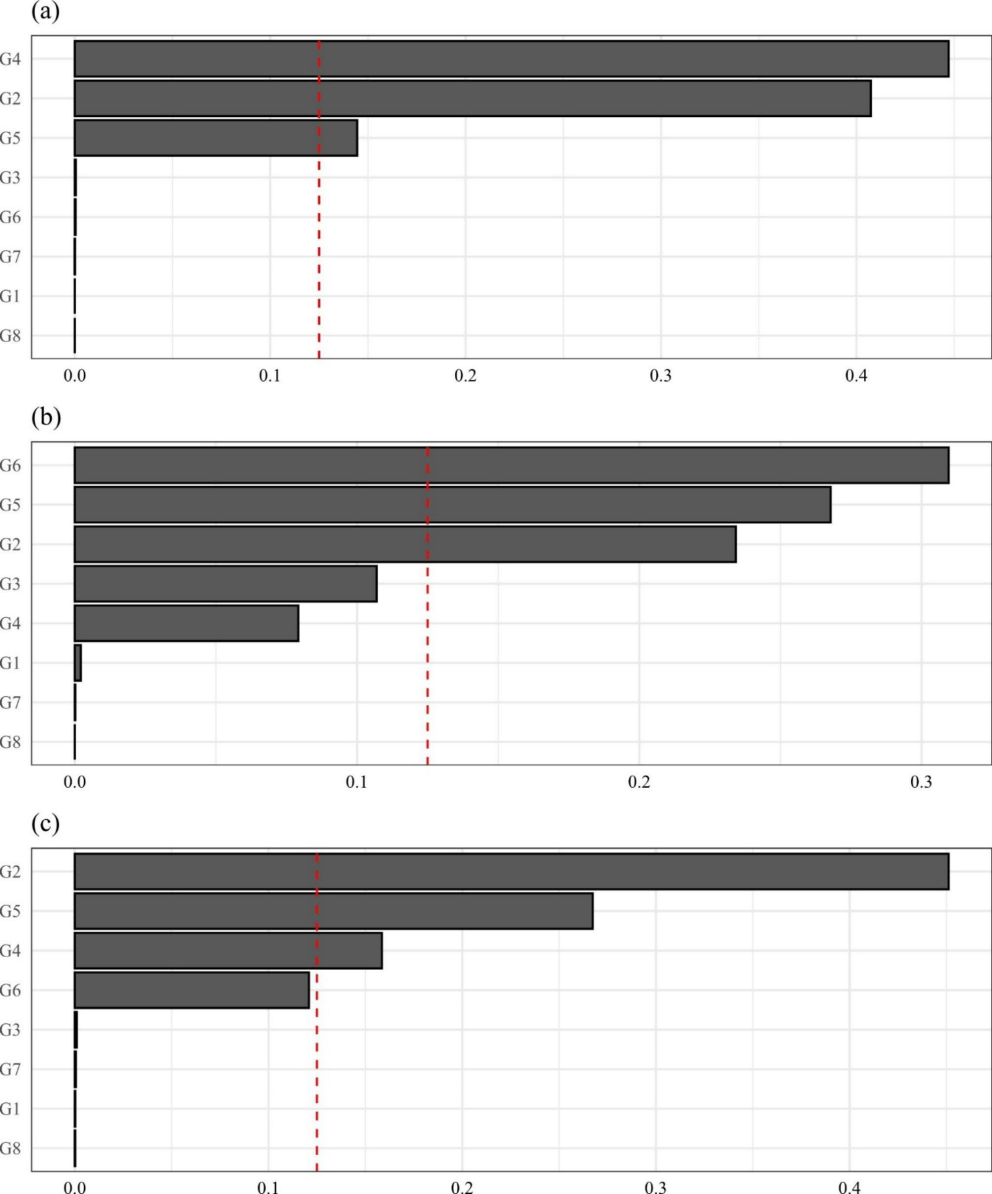
The weights assigned to each PM_2.5_ source group from WQS models on identified source groups. (a): all non-accidental mortality; (b): cardiovascular diseases related mortality; (c): respiratory diseases related mortality; red dash line: reciprocal of the number of PM_2.5_ components in the mixture

The weights of each source group contributing to the association with three outcomes are presented in Table 4; Fig. 2. Compared to the sums of the weights estimated from the WQS models on components, the source group with highest weights for each mortality outcome were still the same as non-tailpipe traffic source, secondary particles from power plants, and biomass burning. Additionally, the weights for tailpipe traffic source get higher and weights for heavy fuel oil combustion get lower. With higher sum weights for tailpipe and non-tailpipe traffic sources, the WQS models on source groups indicated more important contribution from traffic particles to the cumulative associations.

### Total PM_2.5_ Mass Analysis and single component models

The total PM_2.5_ mass analysis results are presented in Table 5. We found similar pattern of associations compared to the cumulative associations estimated from WQS models: positive associations were observed between total mass with all three outcomes, with mortality rate ratio for cardiovascular mortality (RR = 1.1089, 95% CI 1.0981–1.1199 per IQR increase) of greater magnitude than all non-accidental mortality (RR = 1.0369, 95% CI 1.0292–1.0447) and respiratory mortality (RR = 1.0754, 95%CI 1.0598–1.0911).

**Table 5:** Associations between total PM_2.5_ mass and mortality outcomes^a^

Cause-specific Mortality	Mortality Rate Ratio
All Non-accidental Mortality	1.0369 (1.0292, 1.0447)
Cardiovascular mortality	1.1089 (1.0981, 1.1199)
Respiratory mortality	1.0754 (1.0598, 1.0911)

^a^Presented as the mortality rate ratio and 95% confidence intervals for interquartile range increase in concentrations

The results of single component’s individual association estimates are presented in Table S4 in Additional File 1. Positive associations were observed between most of the components and all three outcomes except for Cu & all non-accidental mortality, Cu & respiratory mortality, and Br & all three outcomes. The general patterns across three outcomes were similar to WQS estimated cumulative associations and total PM_2.5_ mass analysis, that the mortality rate ratios were mostly of greater magnitude for cardiovascular mortality compared to the other two outcomes.

## Discussion

We found positive cumulative associations between the PM_2.5_ component mixture with all three outcomes from both WQS models on PM_2.5_ components and source groups. The mortality rate ratios for cardiovascular mortality were of greater magnitude than the other two outcomes. This pattern was also observed in total PM_2.5_ mass analysis and single component models, with positive associations found for total PM_2.5_ mass and most components with all three outcomes. In WQS models on PM_2.5_ components, Fe, OC, and K have the highest weights contributing to the associations, and the sums of weights for the source groups indicating important sources as biomass burning, secondary particles from power plants, and non-tail pipe traffic source. In WQS models on source groups, the most important source group for each outcome were the same, while the weights for tailpipe traffic source get higher.

The 15 PM_2.5_ components included in this study could be considered as markers or surrogates for broader source categories. For the most important components with highest weights identified from WQS models on PM_2.5_ components, K is an indicator for biomass burning, Fe is an indicator for non-tailpipe traffic emissions, OC represents both secondary particles from power plants and tailpipe traffic emissions, Ni represents heavy fuel oil combustion, with other important components SO_4_^2−^ and NO_3_^−^ also represents secondary particles from power plants [18, 26, 53]. We have grouped all 15 PM_2.5_ components in this study into source categories as presented in Table 4. Based on this, we have calculated the sum of weights from WQS models on PM_2.5_ components for each source and also conducted WQS models directly on the mass of source groups. This provided us a clearer view of how the emission from each source contributing to the associations between components mixture and mortality. The high weights found for traffic emissions (include both tailpipe and non-tailpipe) is consistent with the intake fraction studies showing that traffic particles are more likely than average to be deposited in the lung because they are emitted closer to where people walk, live, and work [54, 55]. Therefore, the traffic particles may be more possible to induce adverse health outcomes and appear to have important contribution to the cumulative associations in WQS models, even if toxicity per unit concentration was the same. Besides the traffic emissions, the biomass burning and the secondary particles from power plants, which is mostly coal combustion, are also identified as important source groups. For the biomass burning, its marker component K has the highest weights among all components, although the mass of K is a small fraction of the mass of biomass combustion emissions. For the secondary particles from power plants, SO_4_^2−^ is the major marker with the largest individual mortality rate ratio per IQR increase among the 5 major components.

The unexpectedly high weights for K estimated from WQS models is another issue of attention, which also indicate a need of further exploration of this component. This could be the result from possible measurement errors, residual confounding, and some other unclear reasons specifically related to K. In the future, it is worthwhile to conduct deeper investigation into it.

Several previous studies have also examined the health links of PM_2.5_ emitted from different sources. Most of these studies have identified the association between long-term exposure to traffic-related particle components with adverse health outcomes, like mortality [56, 57], coronary heart disease[57], ischemic heart disease and stroke[58]. A nationwide cohort study in the U.S. reported a strong association between coal combustion particles with ischemic heart disease mortality [59]. Our study results suggest that both biomass burning and traffic-related emissions (both tailpipe and non-tailpipe) play an important role in PM_2.5_’s association with mortality. These results may also suggest general linkages between sources indicative of combustion related activities and mortality since it is in common of all the sources identified with an important roles. Previous studies have also shown strong heterogeneity in PM_2.5_ emission source profiles across different regions [16, 53]. Therefore, our results may also only represent the exposure situation in Massachusetts.

There is heterogeneity in previous studies examining the associations between long-term exposures to PM_2.5_ components and mortality. Several multicity studies based on the ELAPSE (Effects of Low-level Air Pollution: A Study in Europe) and ESCAPE (European Study of Cohorts for Air Pollution Effects) projects have examined Cu, Fe, K, Ni, Si, V, and Zn. Some earlier studies have found null associations between these components and mortality with heterogeneities across different regions [18, 19], but a recently published study based on the ELAPSE project authors have reported positive associations between Cu, Fe, K, Ni, Si, V, Zn and mortality [17]. Some other studies have also supported associations between Zn, Si and mortality [21, 22, 26]. For Cu, Fe, and K, the previous studies have yielded more heterogenous results, with some presenting positive associations [21, 26, 27] and some presenting null findings [22, 23, 27]. For the major components, the heterogeneity in previous studies still exist. A paper based on California Teacher Study has reported positive associations between EC, OC, SO_4_^2−^, NO_3_^−^ with all-cause mortality and cardiopulmonary mortality[21]. However, there are also studies reported null associations between those components with mortality [22, 23]. Some studies have examined NO_3_^−^, SO_4_^2−^, NH_4_^+^ together as secondary inorganic aerosols (SIA) and shown associations with cardiovascular mortality but not with all-cause and respiratory mortality [24, 25]. Our study also reported a general pattern of greater mortality rate ratio magnitudes for cardiovascular mortality than all non-accidental and respiratory mortality. The heterogeneities in previous PM_2.5_ components studies may come from different levels of correlation amongst the individual components, different component profiles, meteorological conditions, population characteristics, prediction accuracies of exposure models, and the infiltration of air indoor[13]. The overall exposure differences between locations, different exposure measurement method and data availabilities, different demographic characteristics, housing stock, and commuting patterns may also contribute to the heterogeneities. With these issues, the generalizability of the studies will be limited.

This is one of the few studies addressing health links of PM_2.5_ components and thus inevitably bears several limitations. The first limitation concerns our mortality rate estimates. We have used the annual total population of each Census tract (> age 18) to approximate the annual person-year at risk at that Census tract. The total population data from 2001 to 2008 was linearly interpolated from U.S. Decennial Census and American Community Survey estimates, which is also an approximation of the actual population counts. These approximations could lead to inaccuracies in the mortality rate estimates. Second, although we have applied a high spatial resolution prediction model, there could still be exposure measurement errors come from lack of indoor air pollution measurements, commuting across different Census tracts, lack of information on residential history, and the residual prediction error of our model. We would expect this kind of exposure measurement error to be non-differential, and lead to a bias towards null though [60]. Moreover, the racial make-up of our study population is also a limitation to the results generalizability. Our study population is highly homogeneous with 93% non-Hispanic white, while non-Hispanic Whites are only about 70% in the whole population of Massachusetts and about 60% in the whole U.S. according to the most recently data [61, 62]. Non-Hispanic Whites are older than the population as a whole, hence their higher representation among all deaths. Different racial make-up could be linked with different exposure patterns [63]. Therefore, there may be vulnerable populations that could not be well represented by this study.

There are also some limitations in our analysis models. In WQS models, we have not incorporated the non-linearities of confounders in the WQS models, which could be a source of residual confounding. The interactions among different components in the mixture also have not been considered in WQS models [29]. Moreover, the source group we have used was based on a priori identified categories but not a formal source apportionment, which may have limitation in accuracy. Our secondary analysis of individual component models also has limitation in controlling the confounding by total PM_2.5_ mass and other co-existing components [64]. It is worthwhile to explore this further in future studies.

This study also has advantages. In comparison with previous studies, one major advantage of our study is the application of a new exposure prediction model with high spatial resolution and prediction accuracy covering a long period from 2000 to 2015. These models have yielded very high prediction accuracies. Therefore, although there were still residual exposure measurement errors, we could still capture the exposure with less errors than most previous simpler air pollution models. Another advantage of our study is that we have stronger study power by incorporating all deaths in Massachusetts from 2000 to 2015. Many previous studies on long-term PM_2.5_ components exposures were cohort studies that identified several thousand deaths during the follow-up [18, 23, 24], or up to tens of thousands of cases [17, 25]. In our study, we have included nearly 800 thousand deaths, which provided a much stronger study power than the previous studies. Finally, we have applied a mixture analysis in our study treating the PM_2.5_ components as a mixture people simultaneously exposed to. Using the WQS models, we could reduce the impact of collinearity of highly correlated components, and better estimate the cumulative associations and the relative importance of each component contributing to the associations in mixture analysis settings.

## Conclusion

The WQS results showed strong positive association between the PM_2.5_ components mixture with all three types of mortality, with Fe, OC, and K identified as potential important components, and biomass burning, traffic emission, and secondary particles from power plants as potential important sources having high contribution to the cumulative associations. The mortality rate ratios for cumulative associations were of greater magnitude for cardiovascular mortality than all non-accidental mortality and respiratory mortality. This pattern was also observed in total PM_2.5_ analysis and single component models.

## Electronic supplementary material

Below is the link to the electronic supplementary material.

Supplementary Material 1

## Data Availability

The mortality data has to been obtained from the Massachusetts Department of Public Health, and the applicants must complete the application process on their own to obtain it. The PM_2.5_ components concentration data are generated from the exposure models developed by Heresh Amini (heresh.amini@sund.ku.dk), and the personnel who would like to access this data may can directly contact Heresh Amini. The covariates data are available in the IPUM data service, the gridMET dataset, and the MEDPAR files of the Center for Medicare and Medicaid Services.
