# Supplementary material for: Associations between long-term exposures to airborne PM2.5 components and mortality in Massachusetts: mixture analysis exploration
Source: Environ Health. 2022 Oct 11;21:96. doi: 10.1186/s12940-022-00907-2 (PMC9552465; doi:10.1186/s12940-022-00907-2)
Supplement: Supplementary file 1 — Supplementary Material 1 [file 12940_2022_907_MOESM1_ESM.docx]

**Associations between long-term exposures to airborne PM_2.5_ components and mortality in Massachusetts: mixture analysis exploration**

*Additional File 1*

Tingfan Jin^a^, Heresh Amini^b^, Anna Kosheleva^a^, Mahdieh Danesh Yazdi^a^, Yaguang Wei^a^, Edgar Castro^a^, Qian Di^c^, Liuhua Shi^d^, Joel Schwartz^a^

^a^ Department of Environmental Health, Harvard T.H. Chan School of Public Health, Boston MA, US

^b^ Department of Public Health, University of Copenhagen, Copenhagen, Denmark

^c^ Vanke School of Public Health, Tsinghua University, Beijing, China

^d^ Gangarosa Department of Environmental Health, Rollins School of Public Health, Emory University, Atlanta, GA

**Corresponding author:**

Tingfan Jin

Department of Environmental Health

Harvard T.H. Chan School of Public Health

Boston, MA, United States

[tingfanjin@hsph.harvard.edu](mailto:tingfanjin@hsph.harvard.edu)

**Figure S1a. Massachusetts Census Tract Level Mean Pollutants’ Concentrations Across 2000-2009**


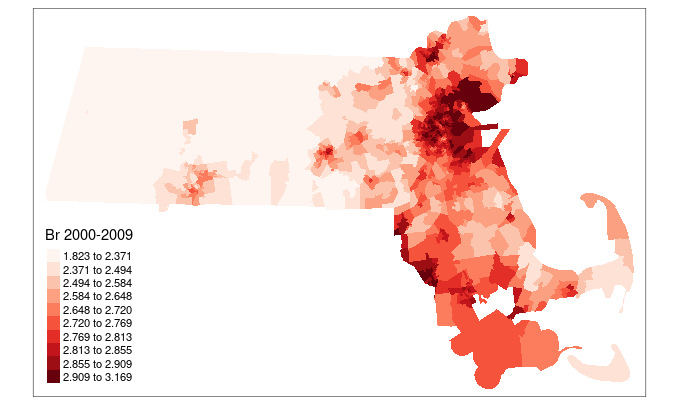

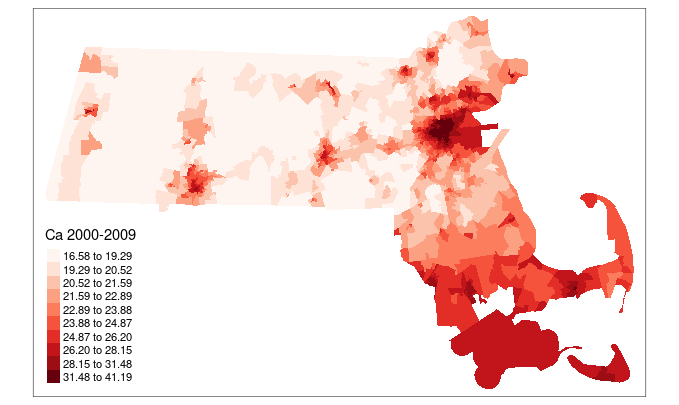


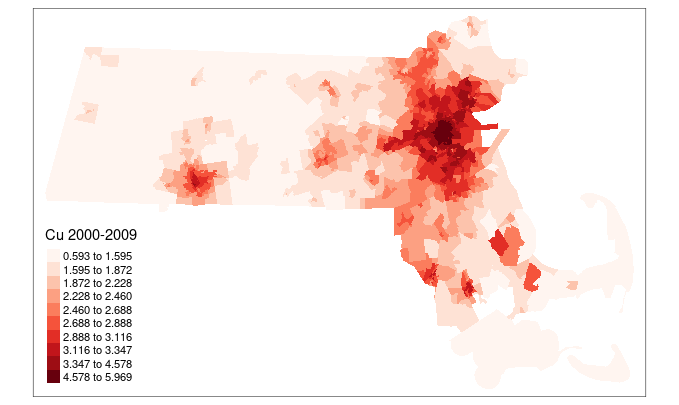

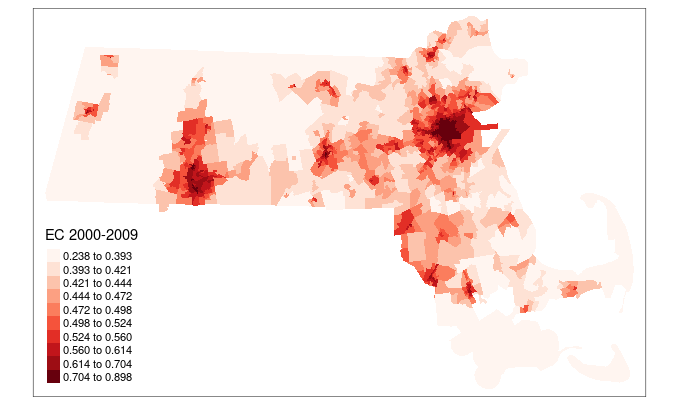


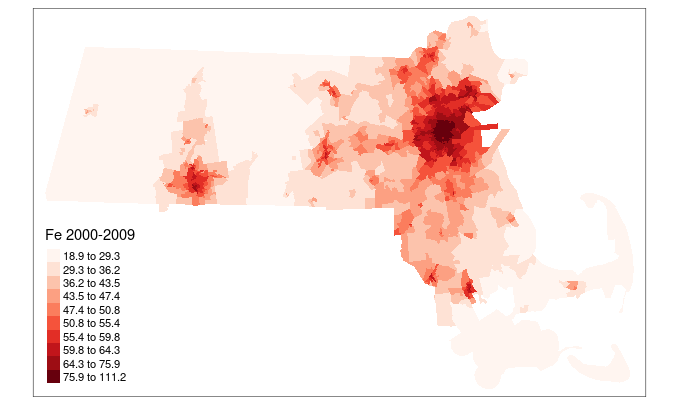

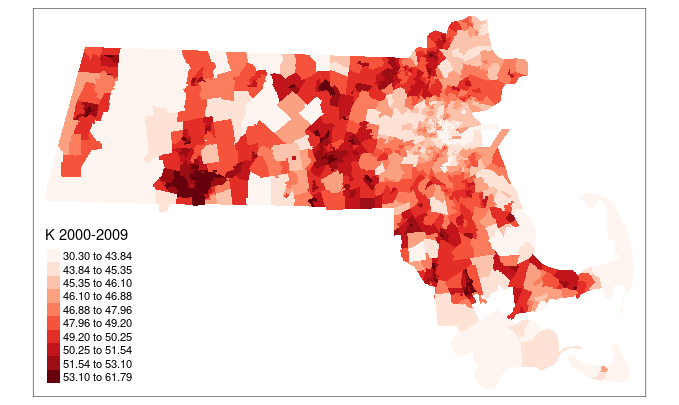


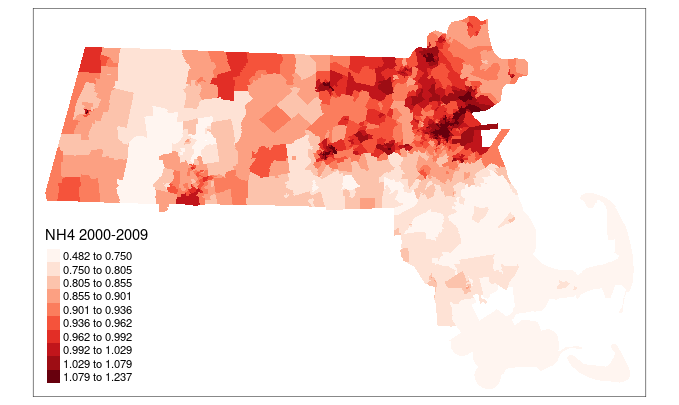

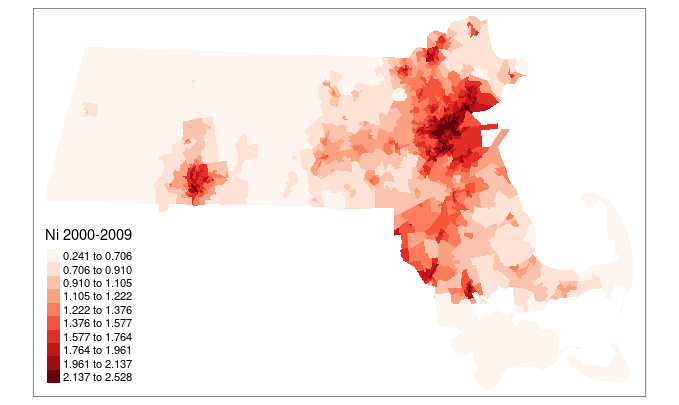


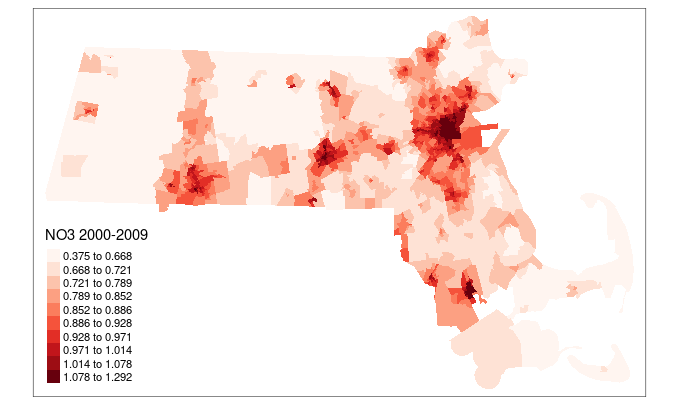

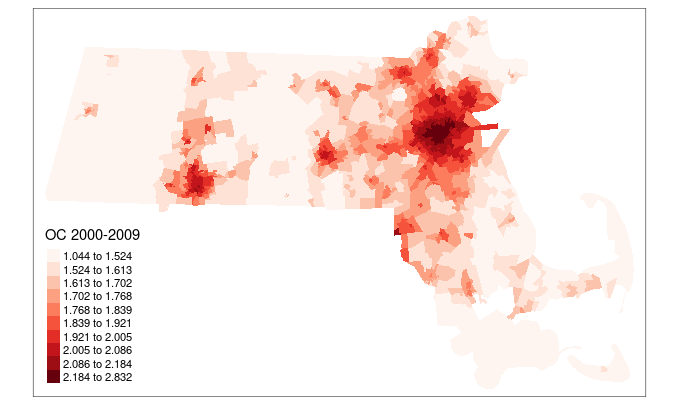


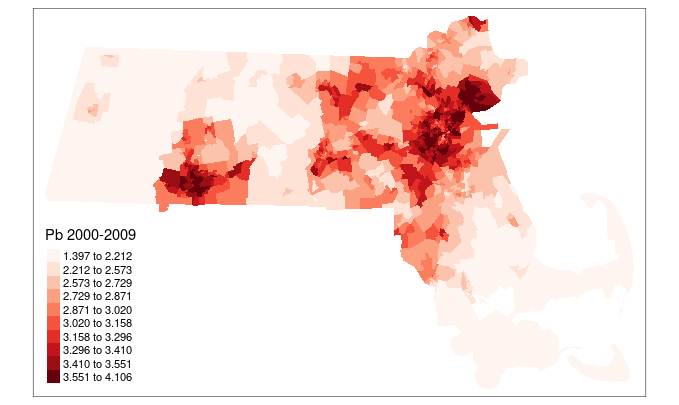

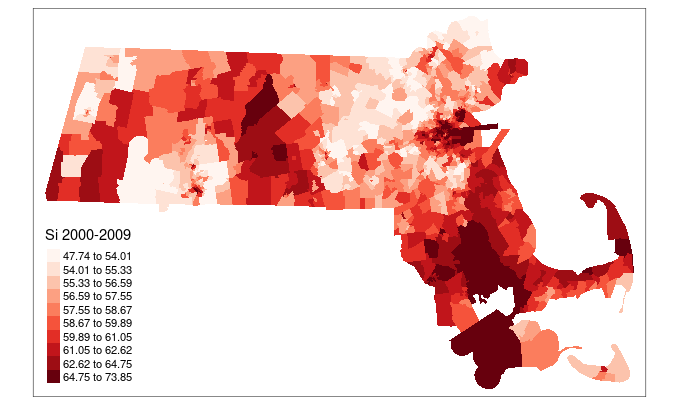


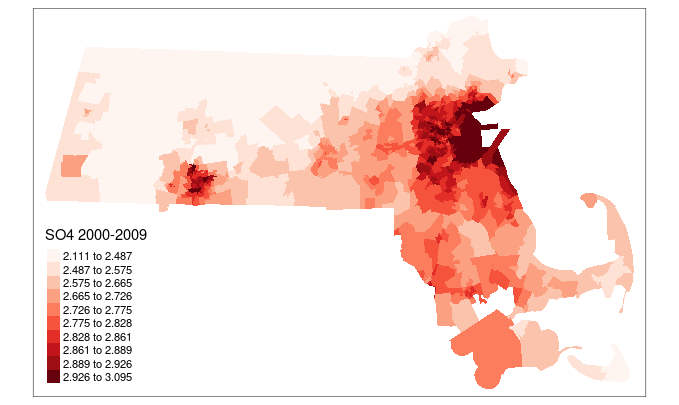

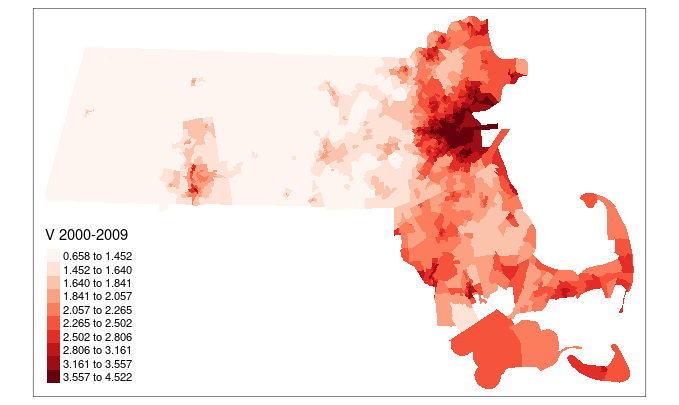


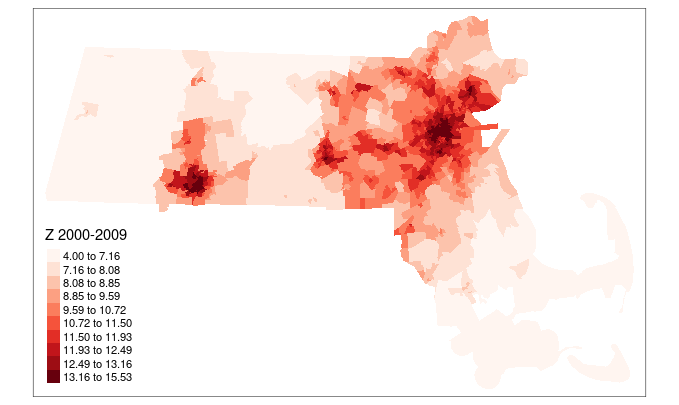


**Figure S1b. Massachusetts Census Tract Level Mean Pollutants’ Concentrations Across 2010-2015**


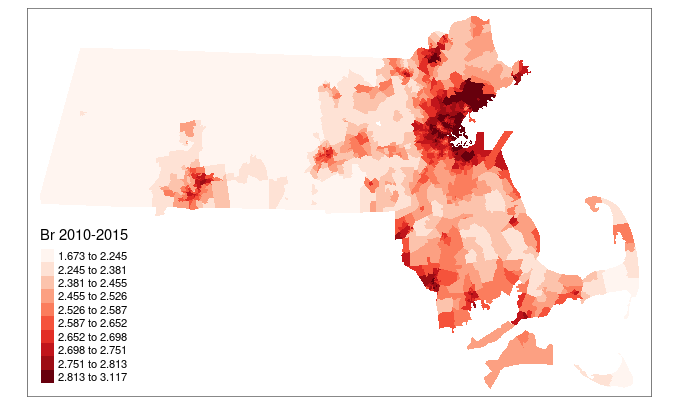

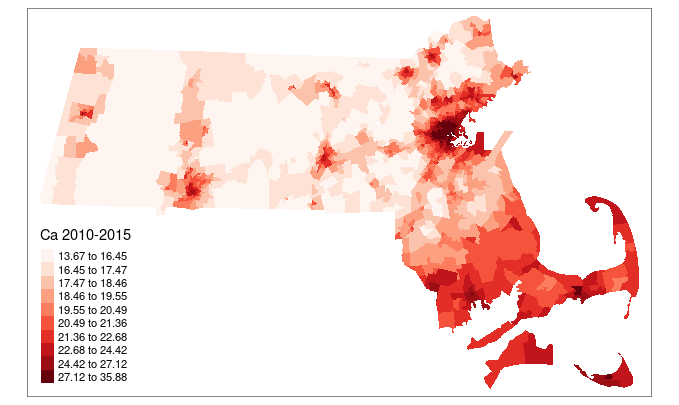


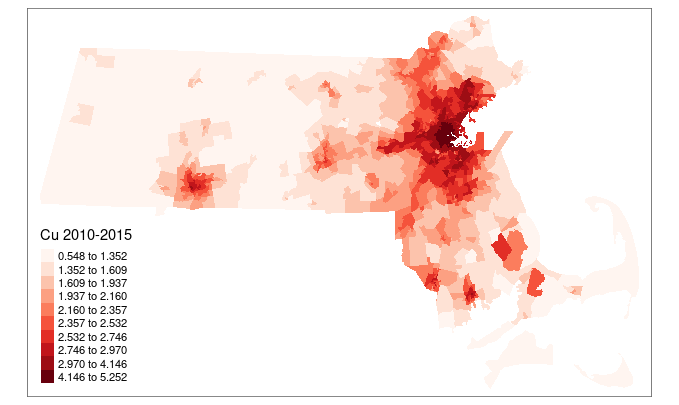

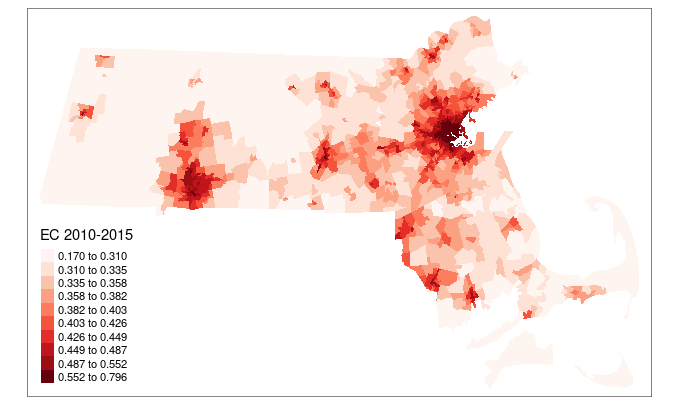


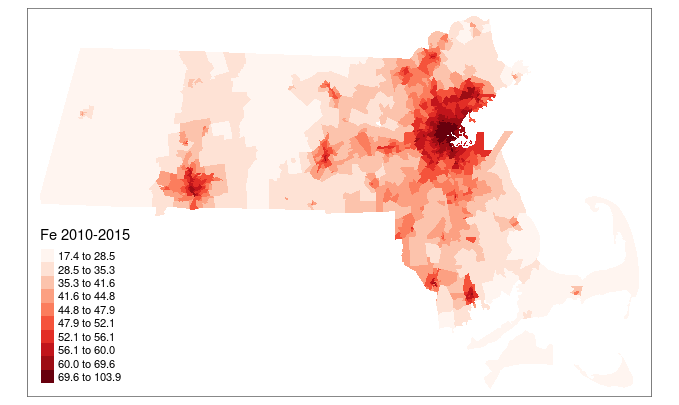

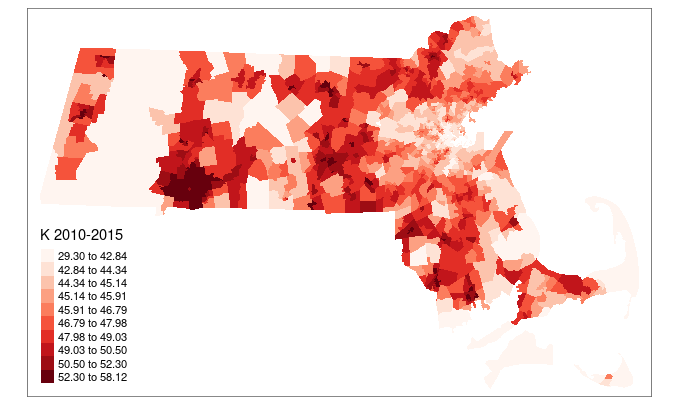


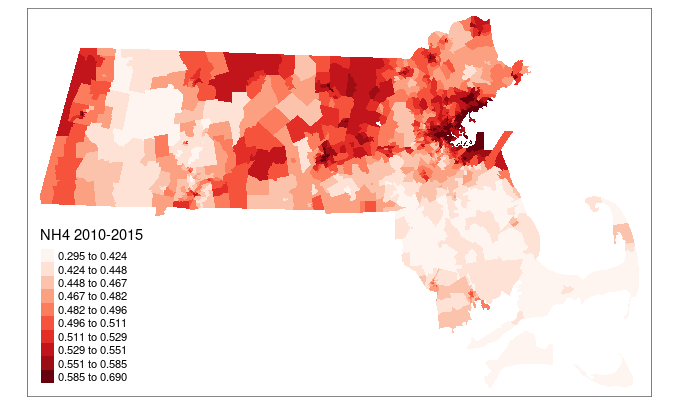

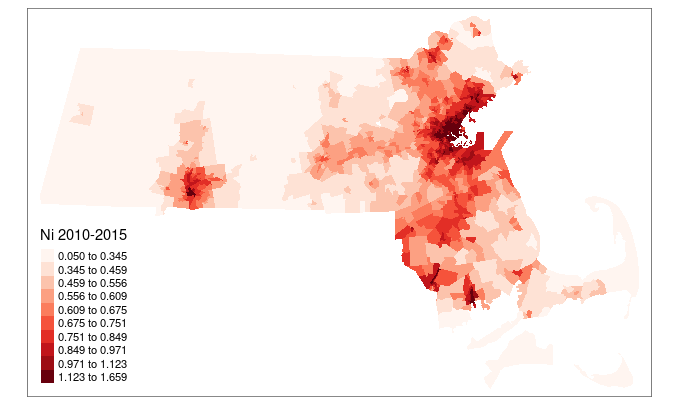


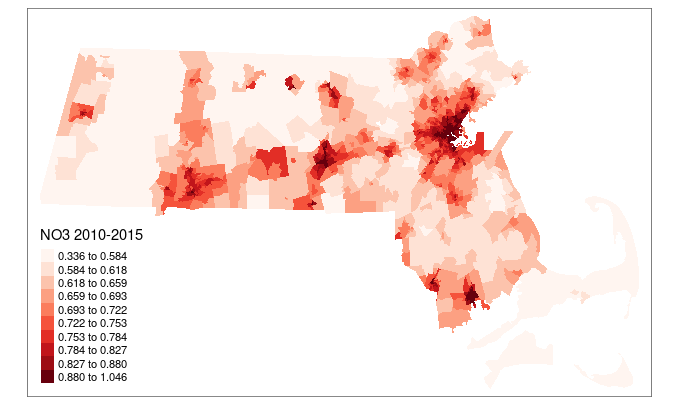

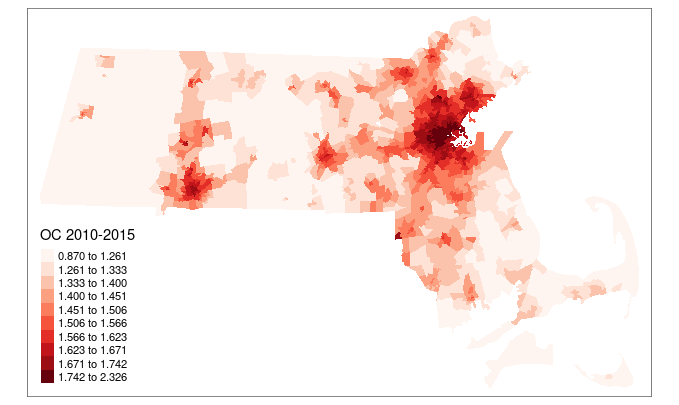


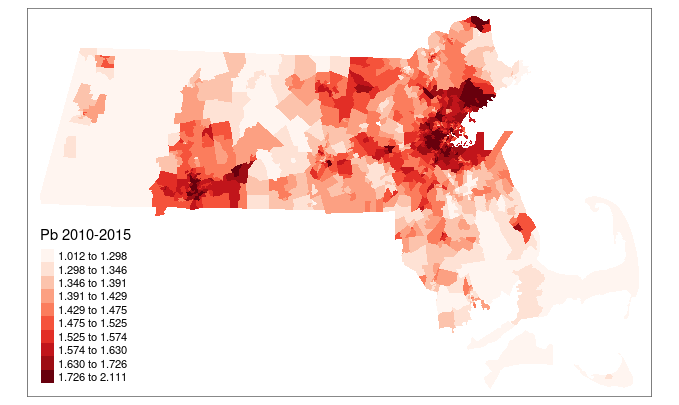

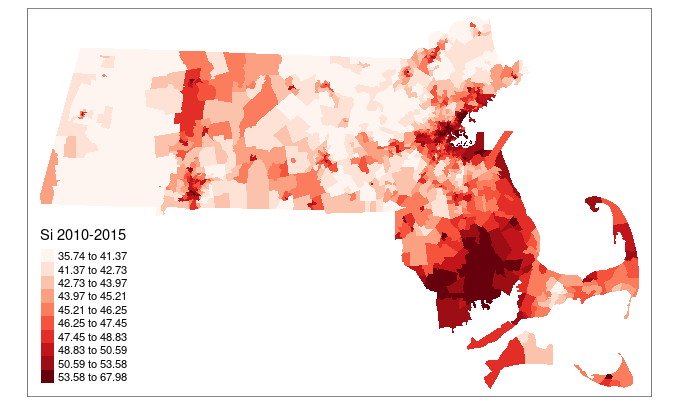


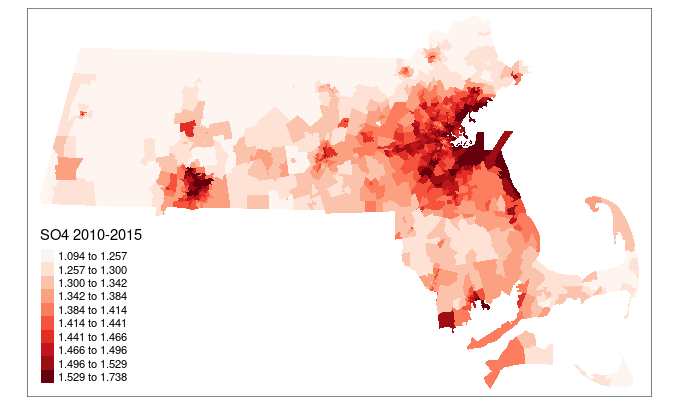

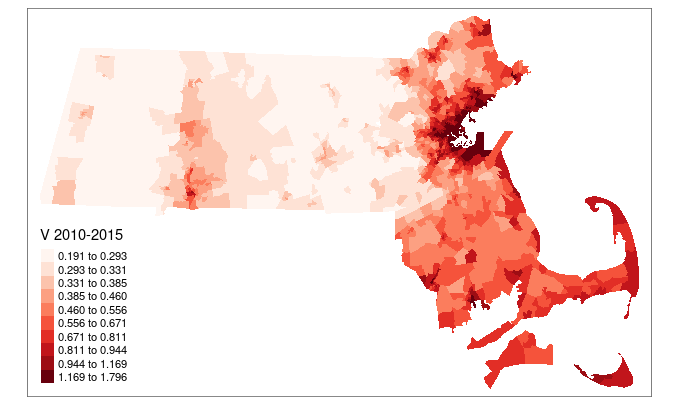


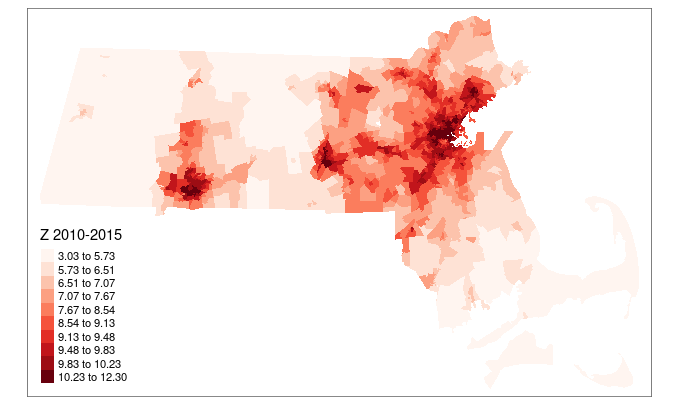


**Figure S2a. Correlation Plot of PM_2.5_ Components**


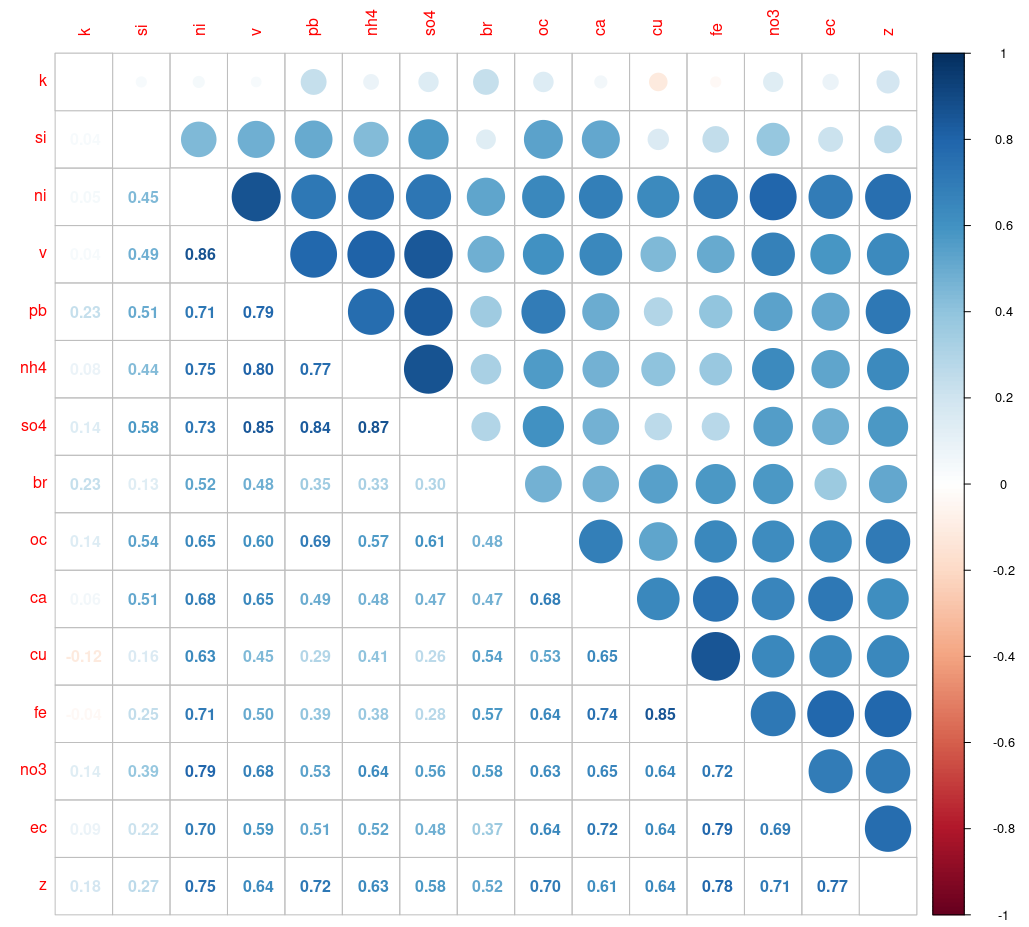


**Figure S2b. Correlation Matrix of PM_2.5_ Components**

**Figure S3. The weights assigned to each PM_2.5_ component from additional WQS models excluding K. (a): all non-accidental mortality, (b): cardiovascular diseases related mortality, and (c): respiratory diseases related mortality (red dash line: reciprocal of the number of elements in the mixture)**


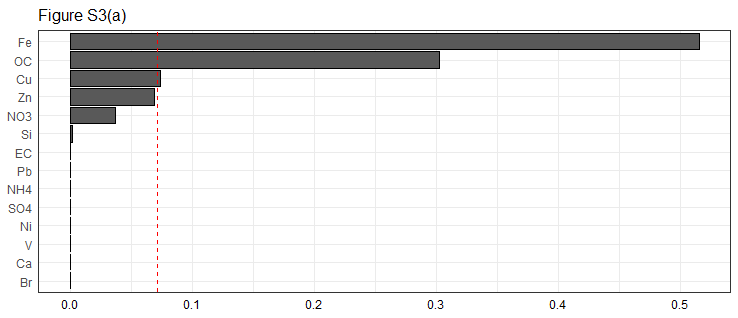


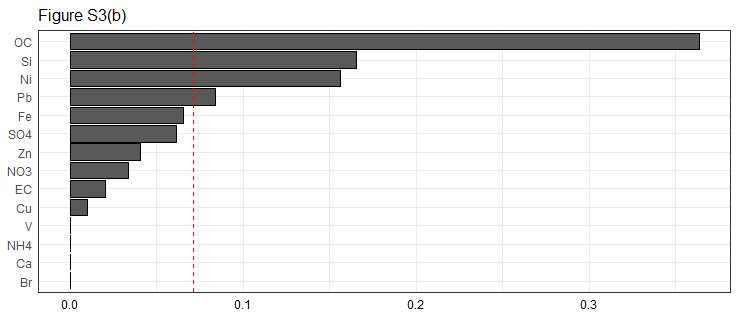


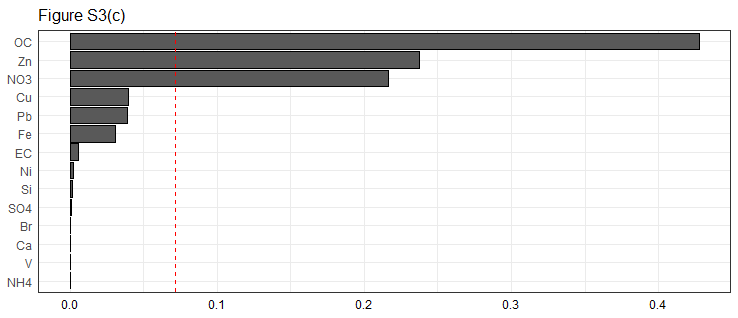


**Table S1. Root-mean-square errors (RMSEs) comparing monitored and predicted annual average PM_2.5_ components concentrations in Massachusetts. RMSEs are calculated from regressing the monitored values against predicted values**

| **PM_2.5_ Components** | **RMSE** | **Mean Concentration in Study** |
| --- | --- | --- |
| ***PM_2.5_ Major Components (μg/m^3^)*** | | |
| EC | 0.09 | 0.48 |
| NH_4_^+^ | 0.09 | 0.76 |
| NO_3_^-^ | 0.07 | 0.82 |
| OC | 0.18 | 1.72 |
| SO_4_^2-^ | 0.28 | 2.21 |
|  |  |  |
| ***PM_2.5_ Trace Elements (ng/m^3^)*** | | |
| Br | 0.18 | 2.63 |
| Ca | 2.86 | 23.30 |
| Cu | 1.35 | 2.66 |
| Fe | 4.99 | 50.80 |
| K | 4.77 | 47.81 |
| Ni | 0.45 | 1.14 |
| Pb | 0.34 | 2.39 |
| Si | 10.15 | 54.34 |
| V | 0.73 | 1.70 |
| Zn | 1.82 | 9.49 |

**Table S2. Mean annual average concentrations of 15 PM­_2.5_ components across all census tracts in Massachusetts from 2000 to 2015**

| **PM_2.5_ Components** | **2000** | **2001** | **2002** | **2003** | **2004** | **2005** | **2006** | **2007** | **2008** | **2009** | **2010** | **2011** | **2012** | **2013** | **2014** | **2015** |
| --- | --- | --- | --- | --- | --- | --- | --- | --- | --- | --- | --- | --- | --- | --- | --- | --- |
| ***PM_2.5_ Major Components (μg/m^3^)*** | | | | | | | | | | | | | | | | |
| EC | 0.67 | 0.54 | 0.44 | 0.52 | 0.53 | 0.57 | 0.51 | 0.50 | 0.48 | 0.44 | 0.45 | 0.46 | 0.44 | 0.38 | 0.38 | 0.39 |
| NH_4_^+^ | 0.79 | 0.93 | 1.03 | 1.00 | 0.99 | 1.06 | 0.96 | 0.94 | 0.81 | 0.72 | 0.59 | 0.61 | 0.46 | 0.44 | 0.47 | 0.43 |
| NO_3_^-^ | 0.81 | 0.84 | 1.00 | 0.96 | 1.02 | 0.90 | 0.84 | 0.91 | 0.74 | 0.75 | 0.64 | 0.74 | 0.75 | 0.69 | 0.74 | 0.77 |
| OC | 2.12 | 2.21 | 2.38 | 1.91 | 1.59 | 1.83 | 1.61 | 1.53 | 1.81 | 1.55 | 1.45 | 1.69 | 1.42 | 1.40 | 1.38 | 1.73 |
| SO_4_^2-^ | 2.68 | 3.22 | 3.04 | 2.99 | 2.93 | 3.04 | 2.66 | 2.51 | 2.41 | 1.88 | 1.56 | 1.70 | 1.42 | 1.36 | 1.23 | 1.14 |
|  |  |  |  |  |  |  |  |  |  |  |  |  |  |  |  |  |
| ***PM_2.5_ Trace Elements (ng/m^3^)*** | | | | | | | | | | | | | | | | |
| Br | 2.57 | 2.54 | 2.83 | 2.81 | 2.57 | 2.95 | 2.85 | 2.68 | 2.57 | 2.34 | 2.22 | 2.42 | 2.52 | 2.47 | 2.70 | 3.01 |
| Ca | 26.22 | 27.47 | 27.84 | 24.90 | 24.23 | 25.15 | 24.26 | 23.30 | 22.71 | 20.32 | 25.23 | 20.96 | 22.94 | 17.14 | 17.09 | 23.96 |
| Cu | 2.14 | 2.31 | 3.21 | 2.86 | 2.43 | 2.96 | 3.42 | 3.41 | 2.89 | 2.28 | 2.72 | 2.47 | 2.58 | 2.13 | 2.10 | 2.76 |
| Fe | 53.07 | 57.86 | 52.38 | 50.71 | 53.00 | 54.73 | 54.32 | 55.15 | 47.89 | 40.83 | 52.71 | 44.99 | 49.81 | 46.60 | 44.84 | 54.72 |
| K | 51.07 | 48.59 | 48.21 | 48.84 | 46.04 | 52.12 | 48.81 | 48.49 | 47.13 | 42.66 | 48.23 | 45.52 | 47.20 | 43.18 | 46.06 | 53.14 |
| Ni | 1.15 | 1.63 | 1.54 | 1.61 | 1.74 | 1.61 | 1.56 | 1.44 | 1.01 | 0.82 | 0.90 | 0.94 | 0.66 | 0.57 | 0.66 | 0.53 |
| Pb | 3.24 | 4.17 | 3.54 | 3.05 | 2.92 | 3.49 | 3.64 | 2.16 | 1.68 | 1.72 | 1.66 | 1.79 | 1.63 | 1.27 | 1.16 | 1.45 |
| Si | 54.12 | 79.55 | 83.87 | 64.65 | 65.26 | 40.95 | 51.13 | 50.65 | 55.31 | 45.19 | 58.16 | 38.04 | 46.71 | 48.03 | 44.07 | 47.09 |
| V | 1.82 | 3.28 | 2.71 | 2.72 | 2.96 | 3.70 | 2.46 | 2.07 | 1.22 | 0.92 | 0.76 | 1.04 | 0.81 | 0.49 | 0.50 | 0.27 |
| Zn | 11.75 | 10.77 | 10.20 | 10.99 | 11.04 | 11.57 | 11.03 | 10.24 | 8.94 | 6.84 | 8.69 | 7.97 | 9.02 | 7.60 | 7.30 | 8.53 |

**Table S3.** **Cumulative associations between PM_2.5_ components and mortality estimated from additional WQS models on all 15 PM_2.5_ components (not on source groups) excluding K^a^**

| **Cause-specific Mortality** | **Mortality Rate Ratios** |
| --- | --- |
| All Non-accidental Mortality | 1.0217 (1.0183, 1.0251) |
| Cardiovascular mortality | 1.0436 (1.0395, 1.0477) |
| Respiratory mortality | 1.0354 (1.0295, 1.0413) |

^a^The mortality rate ratios are per 1 unit increase in the WQS index, which could be interpreted as approximately 1 decile increase in all PM_2.5_ components or the source groups identified for the PM_2.5_ components.

**Table S4. Associations between PM_2.5_ components and mortality estimated from single pollutant quasi-Poisson models. Presented as the mortality rate ratio and 95% confidence intervals for interquartile range increase in concentrations**

| **Components** | **All Non-accidental Mortality** | **Cardiovascular Mortality** | **Respiratory Mortality** |
| --- | --- | --- | --- |
| *PM_2.5_ Major Components* | | | |
| SO_4_^2-^ | 1.0402 (1.0256, 1.0550) | 1.1593 (1.1380, 1.1810) | 1.0878 (1.0579, 1.1187) |
| NO_3_^-^ | 1.0297 (1.0167, 1.0429) | 1.0785 (1.0605, 1.0968) | 1.0579 (1.0317, 1.0848) |
| NH_4_^+^ | 1.0211 (1.0079, 1.0345) | 1.1019 (1.0831, 1.1211) | 1.0438 (1.0171, 1.0711) |
| OC | 1.0381 (1.0281, 1.0481) | 1.0899 (1.0762, 1.1037) | 1.0733 (1.0531, 1.0938) |
| EC | 1.0208 (1.0097, 1.0320) | 1.0580 (1.0429, 1.0734) | 1.0589 (1.0364, 1.0818) |
|  |  |  |  |
| *PM_2.5_ Trace Elements* | | | |
| Zn | 1.0336 (1.0214, 1.0460) | 1.0811 (1.0643, 1.0982) | 1.0757 (1.0508, 1.1013) |
| V | 1.0158 (1.0041, 1.0277) | 1.0860 (1.0696, 1.1028) | 1.0403 (1.0166, 1.0645) |
| K | 1.0381 (1.0283, 1.0481) | 1.0398 (1.0266, 1.0532) | 1.0596 (1.0397, 1.0798) |
| Si | 1.0295 (1.0206, 1.0385) | 1.0814 (1.0692, 1.0938) | 1.0374 (1.0198, 1.0552) |
| Pb | 1.0348 (1.0224, 1.0474) | 1.1145 (1.0969, 1.1323) | 1.0835 (1.0582, 1.1093) |
| Ni | 1.0225 (1.0107, 1.0344) | 1.0903 (1.0737, 1.1070) | 1.0444 (1.0207, 1.0687) |
| Fe | 1.0209 (1.0046, 1.0375) | 1.0601 (1.0375, 1.0832) | 1.0337 (1.0010, 1.0675) |
| Cu | 1.0028 (0.9909, 1.0149) | 1.0294 (1.0129, 1.0461) | 1.0000 (0.9760, 1.0246) |
| Ca | 1.0251 (1.0130, 1.0373) | 1.0883 (1.0713, 1.1056) | 1.0409 (1.0164, 1.0660) |
| Br | 0.9938 (0.9845, 1.0033) | 1.0036 (0.9909, 1.0165) | 1.0056 (0.9868, 1.0247) |

Abbreviations: Cardiovascular Mortality for cardiovascular diseases related mortality; Respiratory Mortality for respiratory diseases related mortality.

**Paragraphs S1. Description of Weighted Quantile Sum Regression Models**

The general idea of Weighted Quantile Sum (WQS) Regression is to: (1) first, summarize the mixture of pollutants into a composite index, where each component’s contribution to the index is weighted based on their relevance to the outcome; (2) second, we use this composite index as the exposure term to fit a regression model estimating the cumulative association between the pollutant mixture and the outcome. In our study, the pollutants’ mixture being studied is composed by the 15 PM_2.5_ components.

To fit the WQS models, the full dataset is first be divided into training set and validation set. The training set is used for the composite index and components’ weights estimation, and the validation set is used for the regression model estimating the cumulative association between mixture and outcome. The proportion of this division is determined by the researcher. In our study, we have used a 50:50 proportion for all WQS models, where 50% of the dataset was split into the training set, and the other 50% was split into the validation set.

**1. WQS index and components’ weights estimation**

The first part of WQS regression is to estimate the composite index, which is the weighted quantile sum (WQS) of the components’ concentration, and the weights of each component contributing to the WQS index.

First, the concentrations of all pollutants are scored into quantiles. By do so, the influence of outliers could be removed. The researchers determine which specific quantile (quartile, quintile, decile, etc.) to be used in the model. In our study, we used deciles, which means that the concentrations of PM_2.5_ components were scored into deciles. Besides the pollutant mixtures, we have also standardized all other covariates, which could be helpful in WQS models.

Second, to estimate the index and weights, several bootstrap samples of the training set is generated. In our study, we have set the number of bootstrap samples as 250 for all WQS models.

Next, the following model is estimated in each bootstrap sample:

$$g\left( Y \right)=\beta_{0}+\beta_{1}\left( \sum_{i=1}^{p} w_{i}q_{i} \right)+\boldsymbol{\beta}_{\boldsymbol{2}}^{\boldsymbol{'}}\boldsymbol{\phi}$$

In this model, Y is the outcome of interest, g is the link function between outcome and regression formula. q_i_ are the quantiles scored for each component i (in our study, deciles), w_i_ are the weights for the component i, **Φ** are the other covariates, β_i_ are the coefficients for each variable. In this model, the parameters to be estimated are βs and the weights w_i_. There are two constraints for the weights: first, each weight is constrained between 0 and 1 (0≤w_i_≤1); second, the sum of all weights equals to 1 ($\sum_{1}^{p} w_{i}$=1). The optimization algorithm used for the parameter estimation has been presented by Carrico et al^[[1]](#footnote-1)^. This article has also been cited in the main text of the manuscript. Moreover, in our study, we have used a log link function and a quasi-Poisson family for the regression models.

In this estimation, the researcher also need to make an assumption of the direction of β_1_: whether it is non-negative or non-positive. In our study, we assumed that the PM_2.5_ components are positively associated with the outcome (mortality), so we determined that the β_1_ to be non-negative. There are two ways in the WQS estimation could be used to fit the constraint of β_1_: (1) constraining the direction of β_1_ in the optimization algorithm when fitting the models in every bootstrap samples; (2) estimate the β_1_ without constraint in every bootstrap samples, and then only use estimated parameters that meet the assumption of β_1_’s direction. In our study, we have used the second approach.

Then, we estimate the final WQS index as:

$$WQS=\sum_{i=1}^{p} \bar{w}_{i}q_{i}$$

In this index, q_i_ are the quantiles scored for each component (in our study, deciles), and $\bar{w}_{i}$ are the weights estimated for each pollutant in the WQS index. These weights are estimated through $\bar{w}_{i}=\frac{1}{B}\sum_{b=1}^{B} w_{i(b)}$, with B as the number of bootstrap samples where the fitted models fulfill the assumption of β_1_, and w_i(b)_ are the weights for component i estimated in these B bootstrap samples. These weights could then be interpreted as the contribution of each pollutant to the cumulative association between the mixture and outcome in the assumed association direction.

**2. WQS regression estimating the cumulative association between pollutants mixture and outcome**

The second part for the WQS regression is using the composite WQS index as the exposure term to fit a regression model estimating the cumulative association between the pollutant mixture and the outcome. This regression model is fitted in the validation set as:

$$g\left( Y \right)=\beta_{0}+\beta_{1}WQS+\boldsymbol{\beta}_{\boldsymbol{2}}^{\boldsymbol{'}}\boldsymbol{\phi}$$

In this model, Y is the outcome, g is the link function, WQS is the WQS index we have already estimated in the first part, **Φ** are the other covariates, and β_1_ is the main coefficient of interest which demonstrates the cumulative association between pollutants mixture and the outcome. In our study, the link function is log and we have set a quasi-Poisson family.

All the WQS regression procedures, including the index and weights estimation and the final WQS regression could are implemented in the gWQS package in R. In our study, we have 3 outcomes of interest (all-cause mortality, cardiovascular mortality, and respiratory mortality), so 3 sets of WQS models have been fitted separately. Moreover, we have also used the sandwich package to estimate the robust confidence intervals for the β_1_ coefficients in the final WQS regression models.

Carrico et al. have discussed the WQS algorithms before, and this paper is a good source for reference regarding the WQS models. This article has also been cited in the main text^[[2]](#footnote-2)^.

1. Carrico C, Gennings C, Wheeler DC, Factor-Litvak P. Characterization of Weighted Quantile Sum Regression for Highly Correlated Data in a Risk Analysis Setting. *Journal of agricultural, biological, and environmental statistics*. 2015;20(1):100-120. doi:10.1007/s13253-014-0180-3 [↑](#footnote-ref-1)
2. Carrico C, Gennings C, Wheeler DC, Factor-Litvak P. Characterization of Weighted Quantile Sum Regression for Highly Correlated Data in a Risk Analysis Setting. *Journal of agricultural, biological, and environmental statistics*. 2015;20(1):100-120. doi:10.1007/s13253-014-0180-3 [↑](#footnote-ref-2)
